# Membrane-Mediated Nanoassembly of Lysozyme–Tannic Acid for Crystallization-Suppressed Nobiletin Delivery: Enhanced Cellular Uptake and Mucus Penetration

**DOI:** 10.3390/biom16020242

**Published:** 2026-02-03

**Authors:** Hongyu Liang, Jiahao Xing, Qiuyue Hou, Luyang Bao, Bin Li, Bin Zhou, Hongshan Liang

**Affiliations:** 1 Cooperative Innovation Center of Industrial Fermentation, School of Life and Health Sciences, Hubei University of Technology, Ministry of Education & Hubei Province, Wuhan 430068, China; 2 College of Food Science and Technology, Huazhong Agricultural University, Wuhan 430070, China; 3 Key Laboratory of Environment Correlative Dietology, Huazhong Agricultural University, Ministry of Education, Wuhan 430070, China

**Keywords:** lysozyme, tannic acid, nobiletin, self-assembly, intracellular transport

## Abstract

To enhance the bioavailability of hydrophobic nobiletin (NOB), this study constructed nanoparticles (LT-NOB) via self-assembly of lysozyme and tannic acid (TA). The multivalent weak interaction network between TA and lysozyme effectively encapsulated amorphous NOB, inhibiting crystallization. The optimized LT-NOB exhibited a size of 212 nm, high encapsulation efficiency (89.5%), and drug loading (47.25%). Cellular uptake was significantly improved, primarily through macropinocytosis, followed by lysosomal escape and endoplasmic reticulum targeting. In Caco-2 and co-culture models, LT-NOB enhanced mucosal permeation by 75% and 50%, respectively, compared to free NOB. This work elucidates a robust strategy for stabilizing amorphous drugs and promoting their intestinal absorption, providing a foundation for advanced nanodrug delivery.

## 1. Introduction

Precise design and modulation of intermolecular interactions are pivotal strategies for enhancing the delivery efficiency of bioactive substances, exerting profound impacts on the development of fields such as pharmaceuticals, functional foods, and nutrient delivery [1]. By directionally regulating non-covalent interactions including hydrogen bonding, electrostatic interactions, hydrophobic effects, and π-π stacking, the programmable modulation of material solubility, stability, and functional activity can be achieved., In food science, the synergistic assembly of biomacromolecules (e.g., proteins, polysaccharides) and functional small molecules (e.g., polyphenols) has emerged as an innovative pathway to overcome the bioavailability bottleneck of hydrophobic functional factors [2]. For instance, lactoferrin-epigallocatechin gallate (EGCG) nanocomplexes significantly enhanced the encapsulation efficiency and photostability of curcumin through a dual electrostatic–hydrophobic interaction mechanism [3].

As a core driving unit for molecular assembly, polyphenols serve as natural molecular assembly ligands due to their unique spatial configuration of phenolic hydroxyl groups and conjugated aromatic ring structures. Among them, the molecular structure of tannic acid (TA), a representative hydrolysable tannin, consists of a glucose core peripherally linked to multiple galloyl groups, endowing it with two key characteristics: (1) Multivalent binding capacity: High-density phenolic hydroxyl groups provide multiple hydrogen bond donor/acceptor sites and enable strong electrostatic interactions with positively charged biomolecules (e.g., proteins) [4]; (2) Coordination chemistry: The pyrogallol units can chelate metal ions such as Fe^3+^/Zn^2+^, enabling self-assembly into pH-responsive metal-phenolic networks (MPNs) [5]. These properties make TA an ideal platform for constructing smart delivery systems. It can encapsulate hydrophobic drugs like paclitaxel via hydrophobic/π-π interactions, and also crosslink with proteins such as bovine serum albumin (BSA) to form core–shell structured nanocarriers for the delivery of functional factors [6].

**Figure 1:**
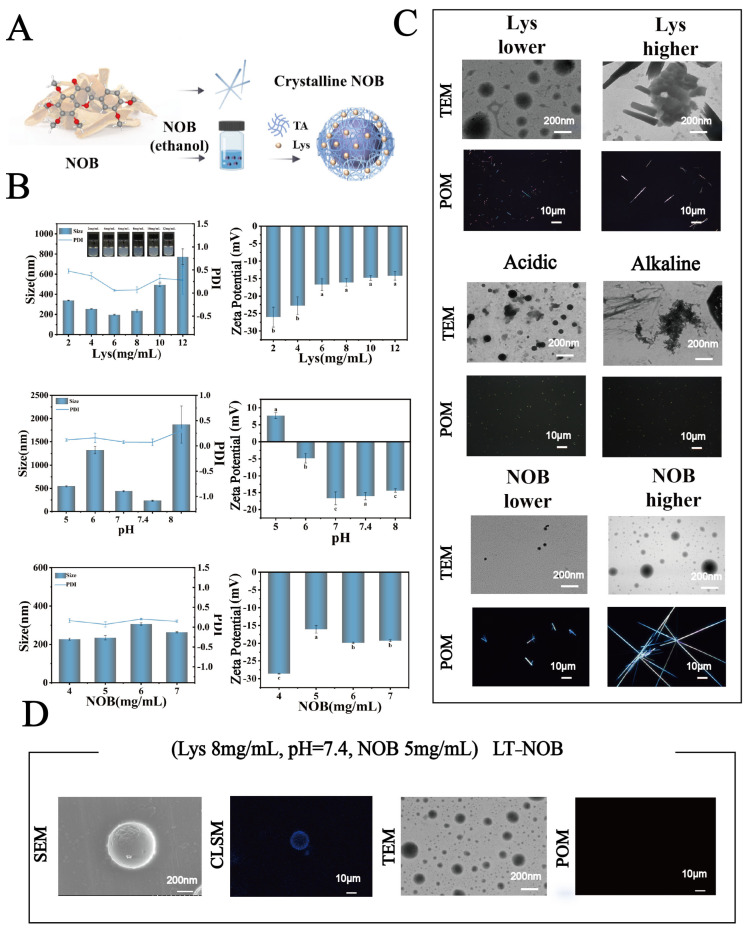
(**A**) Schematic illustration of the system preparation process. (**B**) Particle size, and zeta potential under different conditions. (**C**) Microscopic images of the system under various conditions. (**D**) Microscopic image of the optimized system (*n* = 3). Different letters (a, b, c) indicate statistically significant differences among groups (*p* < 0.05).

However, many existing polyphenol-protein nano-assemblies often rely on a dominant single interaction or require multi-step processing. For example, lactoferrin-EGCG complexes primarily utilize electrostatic and hydrophobic interactions, which may demand precise control over environmental conditions for optimal assembly. Similarly, while TA can effectively crosslink with various proteins like BSA, such systems frequently emphasize the cargo-delivery function of the protein itself, rather than leveraging a synergistic, multi-mechanistic assembly network that concurrently inhibits drug crystallization. In contrast, the lysozyme–tannic acid (Lys-TA) system proposed herein capitalizes on a cascade of well-orchestrated interactions: the strong electrostatic attraction initiates rapid complexation, which is subsequently stabilized by a network of hydrogen bonding and hydrophobic effects. This multi-valent weak interaction paradigm, as detailed in our mechanistic studies (Section 3.3), is crucial for suppressing the crystallization of encapsulated nobiletin. Furthermore, the entire nanoparticle formation is achieved through a straightforward one-step co-assembly process under mild conditions, avoiding the need for chemical crosslinkers or complex purification steps commonly associated with engineered protein carriers, compared to the limitations of traditional protein carriers [7]. Compared to the limitations of traditional protein carriers (e.g., whey protein, human serum albumin, or transferrin), such as complex preparation processes (requiring chemical crosslinking or genetic engineering modification) and high production costs (cumbersome recombinant protein purification procedures), lysozyme exhibits significant advantages: (1) Natural abundance: Widely present in egg white (3.5% content), facilitating industrial-scale extraction. (2) Self-assembly potential: Surface rich in positively charged residues (e.g., lysine/arginine, isoelectric point pI ≈ 11), enabling efficient binding with TA via electrostatic interactions. (3) Functional integration: Inherent lytic activity hydrolyzes peptidoglycan in Gram-positive bacteria, conferring antimicrobial functionality to the carrier [8]. (4) Favorable Safety Profile for Delivery: Lysozyme is an endogenous natural protein that is classified as Generally Recognized As Safe (GRAS) for food applications. It possesses inherently low immunogenicity, which establishes a crucial foundation for its biosafety as a delivery vehicle [9]. Based on this, this study innovatively constructs a lysozyme–tannic acid-nobiletin (LT-NOB) ternary self-assembled nanoparticle system using NOB as the model functional factor, focusing on addressing two key scientific questions (Figure 1A): (1) At the molecular mechanism level: Elucidating the synergistic regulatory rules of Lys-TA electrostatic crosslinking, TA-NOB π-π stacking/hydrogen bonding, and Lys-NOB hydrophobic interactions on the encapsulation efficiency and stability of NOB within the LT-NOB complex; (2) At the delivery behavior level: Revealing the cellular uptake pathways (e.g., clathrin-mediated endocytosis) and intracellular transport kinetics mechanisms of LT-NOB nanoparticles. This research will provide a theoretical paradigm for the development of polyphenol-protein-drug ternary precision assembly systems, accelerating the practical application and translation of nobiletin in functional foods and the pharmaceutical field.

## 2. Materials and Methods

### 2.1. Materials and Reagents

TA (purity ≥ 98%), NOB (purity ≥ 98%), Lys (Purity ≥ 20,000 U/mg), and MOPS (Purity ≥ 99.5%) were purchased from Shanghai Yuanye Bio-Technology Co., Ltd. (Shanghai, China). The human colorectal adenocarcinoma cell line Caco-2 (passages 20–40) was obtained from the Shanghai Cell Bank of the Chinese Academy of Sciences (Shanghai, China). Cells were routinely cultured in DMEM high-glucose medium (Dulbecco’s Modified Eagle Medium; Corning, Tewksbury, MA, USA) supplemented with 10% heat-inactivated fetal bovine serum (FBS; Gibco, Waltham, MA, USA), 1% penicillin-streptomycin dual antibiotics (10,000 U/mL penicillin, 10 mg/mL streptomycin; HyClone, Logan, UT, USA), and 1% non-essential amino acids (NEAA; Gibco, Waltham, MA, USA), using 75 cm^2^ cell culture flasks (Corning, Tewksbury, MA, USA). AssembliesCells were maintained at 37 °C in a humidified incubator (Thermo Scientific, Waltham, MA, USA) with 5% CO_2_.

### 2.2. Construction and Optimization of LT-NOB Assemblies

#### 2.2.1. Preparation of the LT-NOB

To investigate the effect of Lys concentration on the self-assembly behavior of the ternary complex nanoparticles, a series of complexes were constructed by varying the Lys concentration. First, gradient solutions of Lys (2, 4, 6, 8, 10, 12 mg/mL) were prepared, alongside stock solutions of TA (40 mg/mL) and NOB (5 mg/mL). Under continuous magnetic stirring (600 rpm), 1 mL of the NOB stock solution was transferred to a sample vial containing 9 mL of 0.01 M MOPS buffer (pH 7.4). Subsequently, 20 μL of TA solution and 40 μL of Lys solution were rapidly injected sequentially into this system. This resulted in final concentrations in the 10 mL reaction system as follows: NOB at 0.5 mg/mL, TA at 0.08 mg/mL, and Lys ranging from 0.008 to 0.048 mg/mL (corresponding to the 2–12 mg/mL stock solutions, Table 1). Stirring was continued for several seconds after injection, ultimately yielding LT-NOB ternary nanocomplex systems with increasing Lys concentration gradients. Preparation processes under different environmental conditions are in Supplementary Methods.

##### Nanoparticle Size and Zeta Potential Analysis

The particle size and polydispersity index (PDI) of the nanocomplex dispersions were measured using dynamic light scattering (DLS). The zeta potential was also determined. The measured particle size was compared against the size penetration range allowed by the mucosal barrier structure to assess the mucus penetration capability of the nanocomplexes. The zeta potential characterized the surface charge properties, and its value, combined with the PDI, was used to comprehensively evaluate system stability [10].

##### Transmission Electron Microscopy (TEM) Characterization

TEM characterization was performed on the systems. A suspension of the prepared nanocomplex was evenly applied onto 200-mesh ultrathin carbon-coated copper grids using a micropipette and allowed to adsorb for 30 min at room temperature. After natural drying, the morphology was observed using a JEM-2100 (JEOL Ltd., Tokyo, Japan) transmission electron microscope operating at an accelerating voltage of 200 kV. Microstructural information was recorded using a Gatan CCD digital imaging system [11].

##### Polarized Light Microscopy (POM) Observation

The crystalline state of the ternary complex system was analyzed using polarized light microscopy (×50 objective). The sample was dropped onto a glass slide and covered with a coverslip. Substances exhibiting birefringence were observed under cross-polarized light. Free NOB, possessing a distinct anisotropic crystalline structure, displayed bright patches due to its birefringence. In contrast, effectively encapsulated NOB molecules, confined within the nanocomplex, showed reduced crystalline order, appearing as localized dark areas. Thus, polarized light microscopy enabled quantitative assessment of the encapsulation efficiency within the complex system [12].

##### Confocal Laser Scanning Microscopy (CLSM)

To evaluate the dispersion characteristics of the nanoparticles, lysozyme (Lys) was specifically labeled with the Rhodamine B (RhB) fluorescent probe. Specifically: Samples were incubated with 20 μL of 0.1 mg/mL RhB solution in the dark for 15 min, followed by centrifugation at 10,000 rpm for 5 min to remove unbound dye. Leveraging the specific binding between RhB and Lys amino groups, fluorescence signals were captured within the CLSM system using a 543 nm excitation wavelength and a 570–620 nm emission channel. Observations were made using a 60× oil immersion objective [13].

##### Scanning Electron Microscopy (SEM) Analysis

The surface morphology of the ternary complex nanoparticles was characterized using scanning electron microscopy (SEM). 200 μL of the prepared system was transferred using a pipette and evenly applied onto a pre-treated aluminum foil substrate. It was then left undisturbed at room temperature (25 ± 1 °C) for 30 min to allow initial solvent evaporation. To optimize sample morphology uniformity, a layer-by-layer drop-coating method was employed, involving three sequential replenishments (10 min intervals) of solution onto the same area. After complete drying, the aluminum foil was cut into 5 × 5 mm^2^ pieces using scissors. All samples, fixed with conductive adhesive onto SEM stubs, were sputter-coated with gold. Surface morphology was finally characterized under a 5 kV accelerating voltage. Additional methods for characterizing embedding efficiency [14].

##### Ultraviolet-Visible (UV-Vis) Spectroscopy Characterization

UV-Vis spectroscopy was employed to assess the encapsulation efficiency of NOB by Lys-TA. The system was diluted 100-fold with MOPS buffer. UV-Vis spectra were scanned from 200 to 600 nm. The encapsulation effect of the nanocarrier on the drug molecules was characterized by changes in the intensity of the characteristic NOB absorption peak at 329 nm [15].

##### X-Ray Diffraction (XRD) Analysis

To systematically compare the effect of different Lys concentrations on the crystallization behavior of NOB, a blank control group (1 mL NOB solution mixed with 60 μL UPW in 9 mL MOPS buffer) and the Lys-TA-NOB system were prepared. All samples were freeze-dried, ground in an agate mortar to a particle size < 10 μm, and pelletized for XRD testing. Test conditions were set as follows: scanning range 5–60° (2θ), step size 0.02, scan rate 2°·min^−1^, X-ray tube operating voltage 40 kV, current 40 mA. The effectiveness of crystallization inhibition for NOB was evaluated by observing the crystalline peaks [16].

#### 2.2.2. Measurement of Encapsulation Efficiency and Drug Loading

To determine the drug-loading performance of the ternary complex nanoparticles, the free drug content was quantified using a centrifugation-HPLC method. The ternary complex system was centrifuged at 12,000 rpm for 10 min, and the supernatant was collected for free drug quantification. 200 μL of supernatant was mixed with 400 μL of chromatographic-grade acetonitrile (volume ratio 1:2) in a 1.5 mL EP tube and vortexed for 30 s. Quantification of NOB was then performed using an UltiMate 3000 high-performance liquid chromatography system under the following conditions: column: Thermo-C18, 5 μm, 4.6 × 250 mm; mobile phase:acetonitrile-water = 70:30 (*v*/*v*); flow rate: 1.0 mL/min; detection wavelength: 329 nm; column temperature: 25 °C; injection volume: 20 μL [17].

The Encapsulation Efficiency (EE%) and Drug Loading (DL%) were calculated based on the quantitative analysis of free NOB in the supernatant. EE% represents the percentage of total NOB that was successfully entrapped within the nanoparticles, while DL% indicates the mass percentage of encapsulated NOB relative to the total mass of the nanoparticles. The specific calculation formulas are as follows:

EE (%) = [(W_total_ − W_free_)/W_total_] × 100% (1)

DL (%) = [(W_total_ − W_free_)/W_total_NP_] × 100% (2)

where W_total_ is the total mass of NOB added initially, W_free_ is the mass of free NOB detected in the supernatant, and W_total_NP_ is the total weight of the freeze-dried LT-NOB nanoparticles.

### 2.3. Analysis of LT-NOB Interaction Mechanisms

#### 2.3.1. Quartz Crystal Microbalance with Dissipation Monitoring (QCM-D) Analysis

To investigate the binding characteristics of the encapsulation material (Lys-TA) within the complex system (LT-NOB), the interaction kinetics between Lys and TA were systematically analyzed using QCM-D technology. Experiments were performed using gradient concentrations of Lys solution (0.2, 0.4, 0.6, 0.8, and 1.2 mg/mL) with a fixed concentration of TA solution (4 mg/mL), and under different pH conditions (Lys 0.8 mg/mL, TA 4 mg/mL). The specific operational procedure was as follows: First, air was flowed for 20 min to stabilize the baseline, followed by injection of buffer for 30 min to complete background correction. After signal stabilization, TA solution was pumped in at a constant flow rate (100 μL/min) for 30 min, monitoring its binding process to the sensor surface. Finally, different concentrations of Lys solution were introduced. The molecular binding behavior was recorded through the dynamic changes in real-time frequency (Δf) and dissipation factor (ΔD) until the system reached a new equilibrium state [18].

#### 2.3.2. Isothermal Titration Calorimetry (ITC) Analysis

To elucidate the molecular interaction mechanism between Lys and TA, ITC was employed to study their binding thermodynamics. TA was dissolved in MOPS buffers at different pH values (pH 5.0, 7.4, 8.0) to prepare a 200 μM TA solution; Lys was also dissolved in the corresponding pH buffer to prepare a 40 μM solution. All solutions were vacuum degassed (10 min, 25 °C) before data acquisition using a NanoITC system. The syringe was loaded with 50 μL of the polyphenol (TA) solution, and the sample cell was loaded with 300 μL of the Lys solution. The titration program was set to 25 equal-volume injections (2 μL per injection), with an injection interval of 300 s, a stirring speed of 250 rpm, and a constant temperature of 25 °C maintained throughout the experiment. After data collection, the dilution heat background interference was subtracted using NITPIC software (version 1.2.7). Nonlinear regression fitting was performed using a single-site binding model to calculate thermodynamic parameters, including the association constant (Ka), stoichiometry (n), enthalpy change (ΔH), and entropy change (ΔS) [19].

#### 2.3.3. Temperature-Variable Fourier Transform Infrared Spectroscopy (VT-FTIR) Analysis

To investigate the effect of temperature on the molecular conformation of the LT-NOB complex system, temperature-variable Fourier transform infrared spectroscopy was performed on freeze-dried samples. The sample pretreatment procedure was as follows: LT-NOB solutions at different pH values (5.0, 7.4, 8.0) were pre-frozen at −80 °C and then subjected to continuous freeze-drying for 24 h. The freeze-dried powders were then analyzed spectroscopically. Spectral detection used an FTIR spectrometer equipped with a temperature control accessory (resolution 4 cm^−1^, 32 accumulated scans). Experimental parameters were set as follows: temperature was ramped from 37 °C to 100 °C, holding at each target temperature point (10 °C intervals) for 10 min under isothermal conditions. Infrared absorption spectra in the range of 4000–400 cm^−1^ were collected in real-time [20].

### 2.4. Cellular-Level Functional Evaluation

#### 2.4.1. MTT Cytotoxicity Assay

The nanosystem was divided into two groups for cytotoxicity testing: (1) Nanomaterial group: Lys-TA complex stock solution (1 mg/mL) was diluted with complete medium to obtain concentrations of 80, 60, 50, 30, and 15 μg/mL. (2) Free drug group: NOB stock solution (1 mg/mL) was diluted with complete medium to match the concentration series of the nanomaterial group. Caco-2 cells were seeded in 96-well plates and cultured for 24 h. After discarding the original medium, 200 μL of diluted samples were added to each well. Control groups included: (a) Blank control: complete medium only (no cells, no samples); (b) Cell control: cells + complete medium. Following 24 h incubation, the supernatant was removed, and 200 μL of MTT solution (diluted with complete medium) was added to each well. After 4 h of further incubation, 200 μL DMSO was added to dissolve purple formazan crystals through 10 min of shaking [21]. Absorbance was immediately measured at 570 nm using a microplate reader. Cell viability was calculated using Equation (3):

Viability % = (A_sample_ − A_blank_)/A_control_ − A_blank_) (3)

A_sample_: mean absorbance of the drug-treated group; A_control_: mean absorbance of the cell control group; A_blank_: absorbance of the blank control group.

#### 2.4.2. Qualitative and Quantitative Analysis by CLSM and Flow Cytometry

To evaluate the cellular uptake behavior of the LT-NOB nano-drug delivery system, a combined strategy using confocal laser scanning microscopy (CLSM) and flow cytometry was employed. The specific steps were as follows: Caco-2 cell suspensions (density 1 × 10^5^ cells/mL) were seeded into laser confocal-specific culture dishes (35 mm glass-bottom dishes, MatTek) and 6-well plates (Corning), respectively. Cells were incubated at 37 °C under 5% CO_2_ for 48 h until monolayer confluency reached 80–90%. NOB and Coumarin-6 (green fluorescent tracer) were dissolved in absolute ethanol at a concentration ratio of 19:1 to prepare a 5 mg/mL mixed stock solution. Drug-loaded nanoparticles were then prepared according to the method described in Section 2.2.1. The experiment included a free NOB group and a LT-NOB nano-drug carrier group. The NOB concentration in both groups was uniformly diluted to 80 μg/mL using complete culture medium. After removing the original medium from the dishes, 1 mL of the respective treatment sample was added, followed by incubation at 37 °C in the dark for 1, 2, and 4 h. After terminating uptake, the drug solution was discarded. Cells were washed three times with PBS (1 mL per wash) and immediately observed under the confocal laser scanning microscope to visualize intracellular fluorescence distribution (excitation wavelength 488 nm, emission wavelength 500 nm). Cells in the 6-well plates were processed synchronously. After PBS washing, cell suspensions were collected, centrifuged at 1000× *g* for 3 min, the supernatant discarded, and the pellet resuspended in 1 mL PBS. Flow cytometry analysis was then performed using a BD FACSAria III flow cytometer. Untreated cells served as the negative control. The viable cell population was gated based on a biparametric dot plot of forward scatter (FSC) vs. side scatter (SSC). Fluorescence was detected in the FITC channel for 10^4^ cells per sample. Mean fluorescence intensity (MFI) was analyzed using FlowJo v10.8 software [22].

#### 2.4.3. Organelle Co-Localization Analysis by CLSM Imaging

To investigate the subcellular localization characteristics of LT-NOB nanoparticles within cells, Caco-2 cells were subjected to dual-labeling confocal imaging using organelle-specific probes for lys and the endoplasmic reticulum. Caco-2 cell suspension (density 1 × 10^5^ cells/mL) was seeded into glass-bottom dishes and cultured at 37 °C under 5% CO_2_ for 48 h. LT-NOB nanoparticles diluted in complete medium were added to the dishes and incubated for 2 h. After aspirating the nanoparticle-containing medium, cells were washed three times with PBS buffer. Subsequently, 1 mL of HBSS working solution containing the lysosomal red fluorescent probe (LysoTracker Red DND-99, 50 nM) and the endoplasmic reticulum green fluorescent probe (ER-Tracker Green, 500 nM) was added, followed by incubation in the dark for 30 min. After staining, cells were washed three times with HBSS to remove unbound dye and immediately imaged under CLSM. Imaging parameters: lysosome channel (Ex/Em = 577/590 nm), endoplasmic reticulum channel (Ex/Em = 488/515 nm), NOB autofluorescence channel (Ex/Em = 405/450 nm). The degree of nanoparticle co-localization with organelles was assessed [23].

#### 2.4.4. Nanoparticle Endocytosis Mechanism Study

To investigate the cellular uptake mechanisms of LT-NOB nanoparticles, inhibitor blocking experiments were performed to evaluate the contributions of three major endocytic pathways: clathrin-mediated endocytosis, caveolae-mediated endocytosis, and macropinocytosis. The inhibitors used were chlorpromazine (clathrin-mediated endocytosis inhibitor), ethylisopropylamiloride (EIPA, macropinocytosis inhibitor), and filipin III (caveolae-mediated endocytosis inhibitor). Based on preliminary MTT assay results, non-toxic inhibitor concentrations (cell viability > 80%) were selected. Caco-2 cells were seeded in 6-well plates (1 × 10^5^ cells/well) and cultured to 80–90% confluence. Cells were pretreated with inhibitors diluted in complete medium for 30 min, followed by three PBS washes to remove unbound inhibitors. Pretreated cells were then incubated with LT-NOB nanoparticles (80 μg/mL NOB) for 2 h. After drug removal and three additional PBS washes, cells were harvested, centrifuged at 1000× *g* for 3 min, and resuspended in 1 mL PBS. Cellular fluorescence intensity was analyzed via flow cytometry (FITC channel) using untreated cells as negative controls. MFI values were quantified using FlowJo v10.8 software [24].

#### 2.4.5. Investigation of Nanoparticle Intracellular Transport Mechanisms

To explore the intracellular transport mechanisms of LT-NOB nanoparticles, the following inhibitors were added: Brefeldin A (10 μM, inhibitor of endoplasmic reticulum to Golgi transport), Monensin (25 μM, inhibitor of Golgi to plasma membrane transport), Nocodazole (10 μM, inhibitor of microtubule-dependent transport to the plasma membrane), and Bafilomycin A1 (100 nM, inhibitor of lysosomal acidification). Caco-2 cells (density 1 × 10^5^ cells/mL) were seeded into 6-well plates and pre-cultured for 48 h. Cells were then pretreated with complete medium containing the respective inhibitors for 30 min. After washing three times with PBS, cells were co-incubated with LT-NOB nanoparticles (NOB concentration 80 μg/mL) for 2 h. The mean fluorescence intensity (MFI) of the cells was analyzed using FlowJo v10.8 [25].

#### 2.4.6. Establishment of Caco-2 Monolayer Model

##### Transmembrane Accumulative Permeation and Apparent Permeability Coefficient (Papp) Measurement

Caco-2 cell suspension (1 × 10^5^ cells/mL) was seeded in Transwell inserts (apical chamber: 0.5 mL; basolateral chamber: 1 mL DMEM). Cells were cultured dynamically for 21 d at 37 °C, 5% CO_2_, with daily medium replacement. Transepithelial electrical resistance (TEER) was monitored using a Millipore meter: probes were sterilized with 70% ethanol, balanced in PBS for 5 min, and readings were taken. Monolayer integrity was confirmed when TEER stabilized at 600–800 Ω·cm^2^ for 5 consecutive days. Free NOB or LT-NOB nanoparticles (NOB concentration: 80/50 μg/mL) diluted with HBSS were added to the apical chamber (0.5 mL), while the basolateral chamber contained 1 mL HBSS. After 0.5, 1, 1.5, or 2 h incubation, 100 μL samples were withdrawn from the basolateral chamber and replaced with fresh HBSS. Samples were mixed 1:1 with acetonitrile for HPLC analysis. Accumulative permeation (Q_cumulative_) and Papp were calculated using Equations (4) and (5):

(4) $$Q_{\mathrm{cumulative}}\;\mathrm{permeated}\;\mathrm{amount}\;=\frac{\mathrm{VSn}}{\mathrm{VR}}\;\sum_{i=0}^{n-1}\mathrm{Ai}\;+\;\mathrm{An}$$

Q_cumulative_: Corrected cumulative permeation amount at time point n (μg); Ai: The cumulative quantity at the i-th time point or step (e.g., the osmotic amount after each fluid replenishment); The total cumulative quantity from the initial time (i = 0) to the n − 1-th step; An: The baseline cumulative quantity at the n-th step (e.g., the osmotic amount corresponding to the directly measured drug concentration, unit: µg or µmol); VSn: The sample volume at the n-th step (e.g., the actual volume after fluid replenishment, unit: mL); VR: The total volume of the receiving solution (e.g., the volume of the Transwell receiving chamber, unit: mL [26].

(5) $$\mathrm{Papp}=\;\left(\mathrm{dQ}/\mathrm{dt})/(A\;\times \;C_{0}\right)$$

dQ/dt: Drug transport rate, A: membrane surface area, C_0_: initial drug concentration.

##### Investigation of Caco-2 Monolayer Transport Mechanisms

To elucidate the transmembrane transport characteristics of nanoparticles, active/passive transport mechanisms were differentiated using low-temperature inhibition and P-glycoprotein (P-gp) functional blockade assays. The regulatory role of P-gp efflux on drug absorption was also evaluated. Caco-2 monolayers were co-incubated with LT-NOB nanoparticles (NOB concentrations: 80/50 μg/mL) under physiological conditions (37 °C) and energy-dependent transport inhibition conditions (4 °C) for 2 h. In parallel experiments, apical chambers were pretreated with 50 μM verapamil (P-gp inhibitor, diluted in HBSS) at 37 °C for 30 min prior to nanoparticle addition. Basolateral drug concentrations were quantified by HPLC, and apparent permeability coefficients (Papp) and cumulative permeation amounts (Q_cumulative_) were calculated using Equations (2) and (3). Transport efficiency and permeability were compared between temperature-controlled and P-gp-modulated groups to assess mechanistic contributions [27].

#### 2.4.7. Establishment of Caco-2/HT29-MTX Co-Culture Model

To simulate the physiological complexity of the intestinal epithelial barrier, a Caco-2 and HT29-MTX co-culture model was established. Caco-2 cells and HT29-MTX cells were routinely passaged in complete culture medium, respectively. Prior to co-culture experiments, the two cell types were mixed at a 9:1 ratio (Caco-2/HT29-MTX). The density of the mixed cell suspension was adjusted to 1 × 10^5^ cells/mL and seeded onto the apical side of Transwell inserts (0.5 mL/well). Complete medium (1.5 mL) was added to the basolateral compartment. Cells were cultured dynamically at 37 °C under 5% CO_2_ for 21 d, with the medium changed daily. Monolayer integrity was monitored by measuring TEER. The model was considered successfully established when TEER values stabilized between 600 and 800 Ω·cm^2^.

##### CLSM Visualization of Caco-2/HT29-MTX vs. Caco-2 Monolayers

To compare the drug permeation characteristics between the Caco-2 monolayer and the Caco-2/HT29-MTX co-culture model, the permeation behavior within Transwell inserts was visualized using CLSM. The fluorescently labeled LT-NOB nanosystem was diluted in HBSS buffer to an NOB concentration of 80 μg/mL. HBSS solution containing the sample (0.5 mL) was added to the apical chamber, and HBSS buffer (1 mL) was added to the basolateral chamber, followed by incubation at 37 °C for 2 h. After incubation, the solutions from both chambers were aspirated. The membrane of the insert was carefully peeled off, laid flat on a glass slide, and used for CLSM observation. The scanning mode was set to Z-stack scanning during imaging.

## 3. Results and Analysis

### 3.1. System Optimization and Stability Analysis

**Figure 2:**
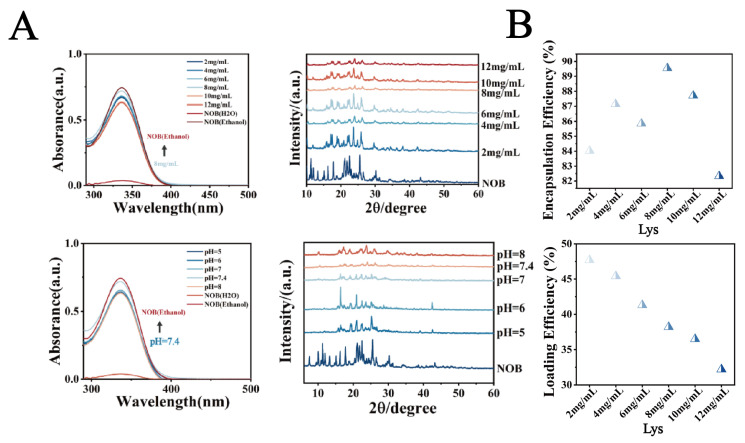
(**A**) UV spectra and XRD patterns. (**B**) Encapsulation efficiency (EE%) of NOB in LT-NOB. Drug loading content (DL%) of NOB in LT-NOB (*n* = 3).

This study systematically investigated the effects of lysozyme (Lys) concentration (2–12 mg/mL), pH (5.0, 6.0, 7.0, 7.4, 8.0), and nobiletin (NOB) concentration (4–7 mg/mL) on the physicochemical properties of nanoparticles and drug encapsulation behavior. As shown in Figure 1B, when the Lys concentration was 8 mg/mL, pH was 7.4, and NOB concentration was 5 mg/mL, the system exhibited optimal particle size distribution characteristics: an average particle size of 212 nm and a polydispersity index (PDI) as low as 0.03, indicating significantly improved nanoparticle monodispersity. The zeta potential remained stable at −17 mV. Furthermore, as shown in Figure 2A, UV-vis spectroscopy analysis revealed that the intensity of the characteristic NOB absorption peak at 329 nm under these conditions most closely resembled its dissolved state in anhydrous ethanol. X-ray diffraction (XRD) patterns further indicated that under these conditions, the characteristic diffraction peaks within the 2θ range of 15–30 exhibited significantly reduced intensity and broadened peak profiles, contrasting with the sharp crystalline diffraction peaks of free NOB, suggesting reduced NOB crystallinity. Morphological analysis (Figure 1C and Supplementary Figures S1–S3) further confirmed the superiority of these conditions: transmission electron microscopy (TEM) observations revealed nanoparticles with a regular spherical shape and uniform size; confocal laser scanning microscopy (CLSM) images showed uniformly dispersed particles. To evaluate drug encapsulation efficiency, polarized optical microscopy (POM) imaging results demonstrated the absence of detectable significant characteristic birefringent crystalline signals of NOB in the system, indicating effective encapsulation of the drug within the nanocarrier.

In contrast, when deviating from these optimal conditions (e.g., Lys concentration lower or higher than 8 mg/mL, pH deviating from neutrality (acidic or alkaline), or different NOB concentrations), the system exhibited characteristics such as increased particle size, PDI > 0.3, and broadening of the particle size distribution peak. Morphological observations also revealed irregularly shaped nanoparticles with poor dispersibility, accompanied by the formation of distinct crystalline precipitates. Notably, to further characterize the microstructure of this optimal system, SEM and CLSM were employed for observation. For CLSM imaging, samples were prepared by depositing a Lys-TA composite film on a substrate of 2 μm polystyrene (PS) microspheres (Figure 1D). SEM images showed that the nanoparticles formed under this condition had smooth surfaces and intact spherical structures; simultaneously, the composite film exhibited continuous and uniform coverage on the PS microsphere surfaces, further confirming the high precision and controllability of the assembly process.

In conclusion, the experimental results demonstrate that under the conditions of Lys concentration 8 mg/mL, pH 7.4, and NOB concentration 5 mg/mL, the system forms nanoparticles with the most regular structure, optimal monodispersity, and achieves effective encapsulation of NOB.

### 3.2. Analysis of Encapsulation Efficiency

Based on optimized parameters (pH 7.4, NOB 5 mg/mL), this study further investigated the regulatory effects of Lys concentration (2–12 mg/mL) on composite membrane thickness and drug-loading performance. As shown in Figure 2B, drug-loading capacity exhibited a concentration-dependent trend, peaking at 8 mg/mL Lys with Drug Loading (DL%) and Encapsulation Efficiency (EE%) reaching 47.25% and 89.5%, respectively. Compared to the 8 mg/mL condition, both DL% and EE% decreased at lower Lys concentrations, while excessive Lys (12 mg/mL) resulted in marginal reductions, likely due to abnormal increases in composite membrane thickness. This strong correlation between membrane thickness and drug-loading performance was attributed to the concentration-dependent self-assembly kinetics of Lys-TA complexes, with 8 mg/mL representing a critical kinetic threshold critical for optimizing drug-loading capacity.

To provide a comprehensive overview of the system’s performance, the key physicochemical characteristics (including particle size, PDI, and zeta potential) and drug-encapsulation metrics (EE% and DL%) under the optimized parameters are systematically compared with those observed under suboptimal conditions in Table 2. This quantitative summary highlights the critical impact of parameter deviations on nanoparticle integrity and reinforces the superiority of the established synthesis protocol (Lys 8 mg/mL, pH 7.4, NOB 5 mg/mL) in achieving high monodispersity and maximal drug loading.

### 3.3. Mechanistic Analysis of LT-NOB Interactions

#### 3.3.1. QCM-D Dynamic Adsorption Behavior Analysis

**Figure 3:**
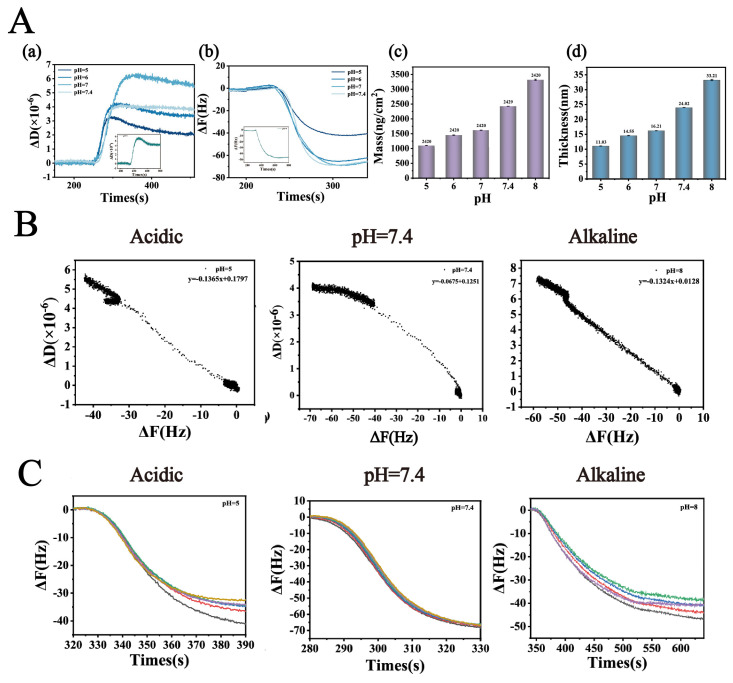
(**A**) (**a**,**b**): Dissipation factor change (ΔD) and Frequency shift (ΔF) of Lys-TA in buffers with varying pH values. (**A**) (**c**,**d**): Membrane thickness and adsorption amount. (**B**) Ratio of dissipation factor change to frequency shift (ΔD/ΔF) as a function of buffer pH, used to characterize the viscoelastic properties of the adsorption layer. (**C**) The multiple curves represent the frequency shifts recorded at different overtones (*n* = 3, 5, 7, 9, and 11). The Y-axis intercept of the ΔF variation curves at low concentrations reflects the compactness of the adsorption layer (*n* = 3).

To deeply elucidate the regulatory mechanism of pH on the interfacial interactions between Lys and TA molecules and its influence on the self-assembly behavior of nanoparticles, this study employed QCM-D to systematically characterize the formation characteristics of Lys-TA composite films. This study systematically investigated the interfacial binding behavior of fixed-concentration Lys (8 mg/mL) and TA (40 mg/mL) across varying pH conditions (Figure 3). QCM-D dynamic monitoring revealed (Figure 3A(a,b)) that ΔF and ΔD values exhibited an initial increase followed by a decrease as pH rose from 5.0 to 8.0: ΔD peaked at pH 7.0 (5.5 × 10^−6^), while ΔF showed maximum negative deflection (−70 Hz) at pH 7.4. These trends indicate enhanced viscoelasticity and adsorption efficiency of Lys-TA composite films under neutral to weakly alkaline conditions, likely attributable to optimized electrostatic complementarity between lysine and tannic acid. At pH 5.0–6.0, TA’s phenolic hydroxyl groups remained highly protonated (pKa ≈ 8.5), resulting in low negative charge density and weak electrostatic attraction with positively charged Lys (pI ≈ 11.0). As pH increased to 7.0, 7.4, partial TA deprotonation significantly strengthened electrostatic and hydrogen bonding interactions with Lys, promoting dense composite film formation. However, at pH > 8.0, reduced Lys surface positive charge (approaching its pI) diminished binding driving forces, leading to decreased assembly efficiency.

ΔD/ΔF correlation analysis further validated the viscoelastic properties of composite films (Figure 3B), with negative ΔD/ΔF ratios observed across all pH conditions, confirming viscous-dominated film behavior. Combined with ΔF sixth harmonic Y-axis intercept analysis (Figure 3C), the minimal intercept (−3 Hz) at pH 7.4 again confirmed optimal film compactness. Sauerbrey model calculations of film thickness and adsorption capacity (Figure 3A(c,d)) revealed that as pH increased from 5.0 to 7.4, film thickness grew from 11.03 nm to 32.21 nm, and adsorption capacity rose from 1107 ng/cm^2^ to 3321 ng/cm^2^. This phenomenon stems from pH-modulated molecular binding mode transitions: at low pH (pH < 7.0), Lys-TA interactions were primarily electrostatic, forming sparse monolayers; under neutral to weakly alkaline conditions (pH 7.0–7.4), TA deprotonation enhanced electrostatic interactions while exposing additional phenolic hydroxyl groups to facilitate synergistic hydrogen bonding and hydrophobic interactions, driving dense multilayer film growth. At high pH, increased TA phenolic hydroxyl dissociation enabled stronger hydrogen bonding with Lys backbone amide groups, compensating for reduced electrostatic interactions through hydrogen bond-hydrophobic force synergy to sustain composite deposition, albeit with looser film structures (reflected by decreased ΔD values).

These findings reveal the dual regulatory role of pH in Lys-TA nanoassembly: moderate charge matching (pH 7.0–7.4) maximizes intermolecular forces for high-density composite film construction, while extreme pH conditions reduce assembly efficiency due to charge repulsion or binding site saturation. This mechanism explains the experimentally observed optimal drug-loading capacity and stability of LT-NOB nanoparticles under neutral conditions [28].

#### 3.3.2. ITC Thermodynamic Parameters and Binding Mode Characterization

**Figure 4:**
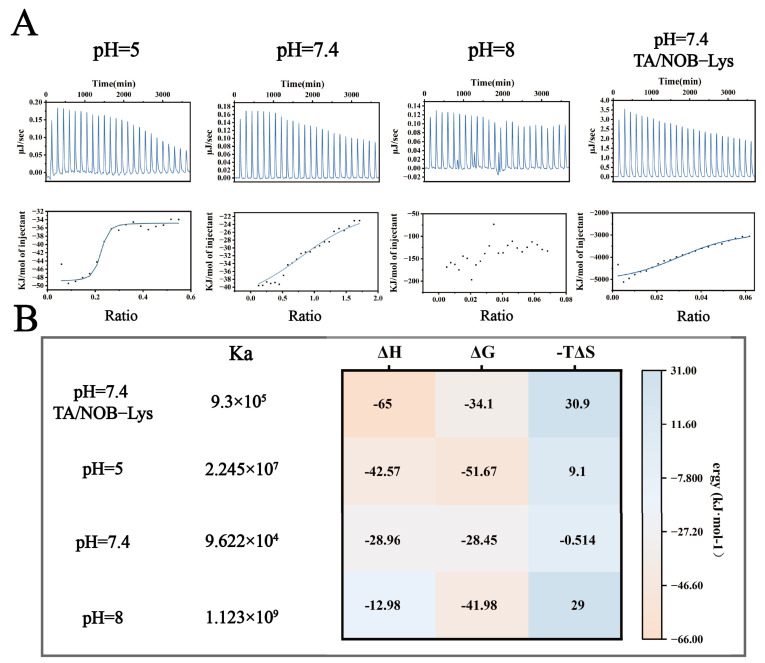
ITC Analysis of LT-NOB Interactions. (**A**): Raw thermogram (upper panel) and corresponding binding isotherm (lower panel) from ITC titration experiments. (**B**): Thermodynamic parameters derived from ITC data fitting: binding constant (Ka), enthalpy change (ΔH), Gibbs free energy change (ΔG), and entropy term (−TΔS) (*n* = 3).

To elucidate the binding mechanisms of Lys-TA nanoassembly under varying pH conditions, this study employed ITC to compare Lys-TA interactions at acidic (pH 5.0), weakly alkaline (pH 7.4), and alkaline (pH 8.0) conditions (Figure 4A,B). At pH 5.0, single-site binding model fitting revealed high binding affinity (Ka = 2.245 × 10^7^ M^−1^) dominated by strong exothermic characteristics (ΔH = −42.57 kcal/mol, −TΔS = +9.1 kcal/mol), indicating dominant electrostatic interactions consistent with partial TA protonation (low negative charge density). At pH 7.4, Ka decreased to 9.622 × 10^4^ M^−1^ with reduced ΔG magnitude (ΔG = −28.45 kcal/mol) and entropy-driven binding (−TΔS = −0.514 kcal/mol), suggesting a transition to multisite synergistic weak interactions (hydrogen bonding/hydrophobic effects). At pH 8.0, disordered titration curves indicated Lys conformational unfolding near its isoelectric point (pI ≈ 11.0), leading to nonspecific multivalent binding. When titrating Lys with TA/NOB mixed solutions at pH 7.4, Ka increased to 9.3 × 10^5^ M^−1^ with significantly enhanced exothermicity (ΔH = −65 kcal/mol), suggesting ternary synergistic binding mediated by aromatic ring conjugation. Substantial entropy loss (−TΔS = +30.9 kcal/mol) reflected increased system rigidity due to NOB immobilization, confirming stable nanoparticle formation.

Combined ITC-QCM-D analysis revealed multiscale assembly rules for the LT-NOB system: under weak alkaline conditions, TA deprotonation enables polyphenol “adhesive” behavior, forming Lys networks through multisite weak interactions (Ka = 9.622 × 10^4^ M^−1^). While such multivalent coordination reduces per-site binding energy (higher ΔG), it significantly enhances overall adsorption capacity, aligning with the “multivalent weak binding enhances macroscopic performance” theory and enabling efficient drug loading [29].

#### 3.3.3. Temperature-Dependent Infrared Spectroscopy Reveals Molecular Conformational Changes

**Figure 5:**
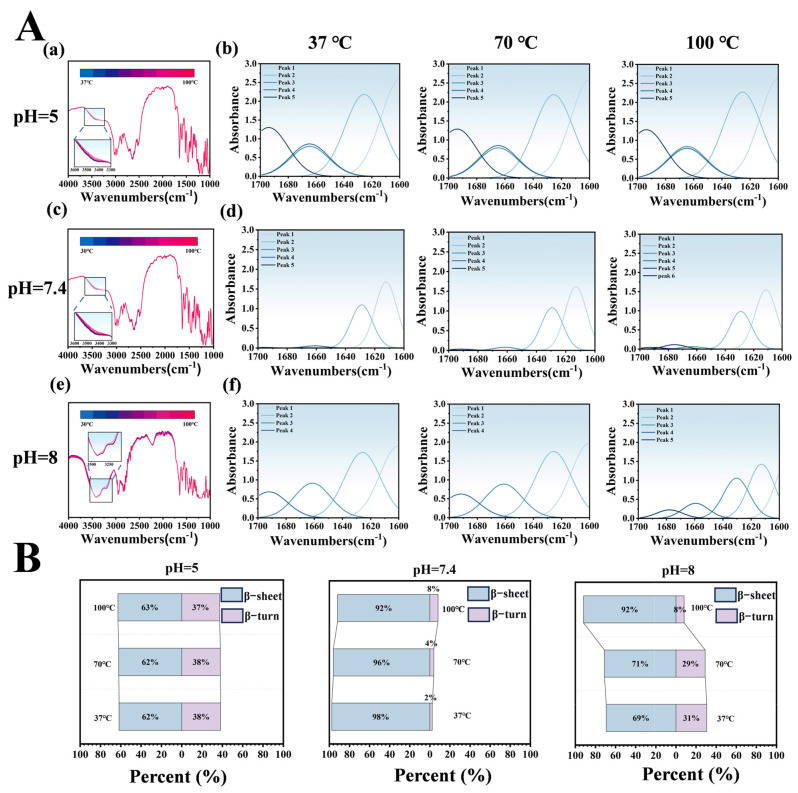
Effects of pH and Temperature Gradients on LT-NOB Secondary Structure Stability (Fourier Transform Infrared Spectroscopy Analysis). (**A**) Variable temperature Fourier transform infrared (VT-FTIR) spectra of LT-NOB under different pH buffer conditions (**a**,**c**,**e**) and corresponding secondary structure fitting analyses (**b**,**d**,**f**). (**B**) Relative percentages (%) of secondary structure components (β-sheets, β-turns) in LT-NOB under varying pH and temperature conditions, calculated from the fitting results in Figure 5A(**b**,**d**,**f**) (*n* = 3).

Temperature-dependent infrared spectroscopy of the LT-NOB system under varying pH and temperature conditions was employed to analyze peak shifts and secondary structure proportions, elucidating intermolecular interactions and thermal stability differences. In Figure 5A(a,c,e), dynamic changes in the hydroxyl/amine stretching vibration peak at 3500 cm^−1^ reflect hydrogen bond network reorganization. At pH 5 and 7.4, blue shifts of ~23 cm^−1^ with increasing temperature indicate hydrogen bond network dissociation-reorganization under thermal perturbation, consistent with literature reports of protein-polyphenol complexes maintaining stable conformations via dynamic hydrogen bonding under acidic to neutral conditions. However, at pH 8, the peak exhibited bimodal distribution with temperature-dependent blue shifts, likely attributable to TA oxidative polymerization-induced structural heterogeneity. Elevated temperatures accelerated TA oxidation, forming quinoid structures that engage in π-π stacking with Lys, particularly evident at pH 8, resulting in bimodal spectral features and blue shifts.

Secondary structure analysis (Figure 5B) revealed significant pH-dependent regulation of LT-NOB conformations. At pH 5, β-turns constituted 62% and β-sheets 38%, with minimal structural changes upon heating, suggesting TA stabilizes Lys β-turns via hydrogen bonding, preserving partial native conformations. At pH 7.4 (physiological pH), β-turns dominated (98%) with negligible β-sheet content (2%). Even at 100 °C, β-turns only decreased to 92% while β-sheets increased to 8%, indicating multivalent Lys-TA bonding forms highly ordered cyclic/helical structures that suppress β-sheet formation and confer exceptional thermal stability. Conversely, at pH 8, initial β-turns (69%) and β-sheets (31%) ratios underwent drastic changes upon heating: β-turns surged to 92% while β-sheets dropped to 8%. This phenomenon likely stems from TA oxidation-induced rigidity loss, where thermal energy disrupts Lys rigid structures, driving β-sheet-to-β-turn transitions.

Infrared and secondary structure analyses demonstrate that pH modulates conformational stability through intermolecular interactions (hydrogen bonding, electrostatic effects), while temperature drives secondary structure transitions via noncovalent bond disruption/reorganization. At pH 5 and 7.4, highly ordered β-turn-dominated structures exhibit superior thermal stability, whereas pH 8 conditions induce structural heterogeneity through TA oxidation, leading to abrupt thermal conformational changes that limit practical applicability [30].

**Figure 6:**
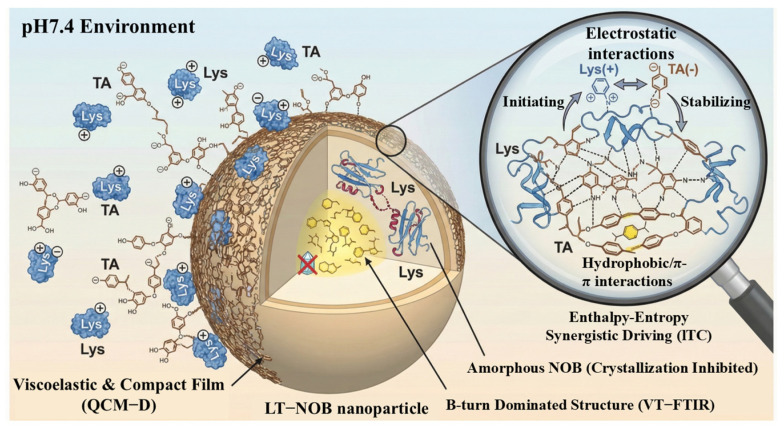
Schematic illustration of the assembly mechanism and structure of LT-NOB nanoparticles at pH 7.4.

Collectively, these spectroscopic and thermodynamic analyses elucidate the multi-scale assembly mechanism of the LT-NOB system. As systematically illustrated in Figure 6, the assembly is initiated by the formation of a viscoelastic and compact composite film under neutral pH (QCM-D), stabilized by a synergistic network of electrostatic, hydrogen bonding, and hydrophobic interactions (ITC), and finally locked into a thermally stable β-turn-dominated conformation that effectively suppresses drug crystallization (VT-FTIR).

### 3.4. Cellular-Level Functional Evaluation

#### 3.4.1. MTT Assay for Cytotoxicity Assessment

To evaluate the biocompatibility of the LT-NOB system, this study measured the cytotoxicity of NOB and Lys-TA carriers against Caco-2 cells at concentrations of 80, 60, 50, 30, and 15 μg/mL using the MTT assay (Supplementary Figure S4). Results showed that even at the highest concentration (80 μg/mL), cell viability exceeded 85% in all groups, confirming negligible cytotoxicity of both Lys-TA carriers and NOB. Consequently, subsequent transmembrane transport studies utilized 80 μg/mL NOB (LT-NOB and free NOB).

#### 3.4.2. Cellular Uptake Characterization via Flow Cytometry and CLSM

**Figure 7:**
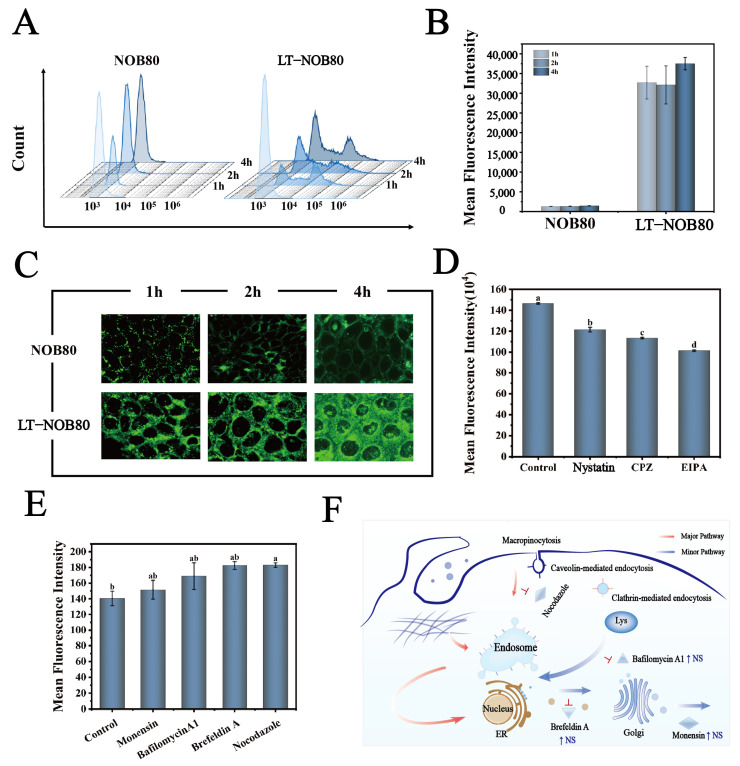
(**A**,**B**) Quantitative comparison of cellular uptake efficiency using flow cytometry. (**C**) Visualization of cellular uptake behavior via CLSM. (**D**,**E**) Flow cytometry-based quantitative analysis of inhibitor effects on nanocarrier cellular uptake. (EIPA, Nystatin, CPZ, Nocodazole, Monensin, Bafilomycin A1, Brefeldin A). (**F**) Schematic Diagram of Cellular Uptake and Transport Mechanism (*n* = 3). Different letters (a, b, c, d) indicate statistically significant differences among groups (*p* < 0.05).

To systematically compare cellular uptake differences between the LT-NOB nanodelivery system and free NOB, quantitative flow cytometry and qualitative CLSM analyses were performed. Flow cytometry revealed significantly enhanced uptake efficiency in the LT-NOB group, with a MFI of 31,580 AU compared to 820 AU in the free NOB group (Figure 7A,B). Notably, MFI increased from 31,580 AU to 36,979 AU (*p* < 0.01) when incubation time extended from 1 h to 4 h, demonstrating marked time-dependent uptake behavior. CLSM qualitative analysis further confirmed progressive enhancement of green fluorescence signals in LT-NOB-treated cells under excitation light, with fluorescent markers predominantly localized to the cytoplasmic matrix (Figure 7C). These findings collectively demonstrate that nanoformulation significantly improves cellular drug uptake efficiency.

#### 3.4.3. Mechanistic Study of Cellular Nanoparticle Uptake Pathways

Based on MTT assay results (Supplementary Figure S4), safe concentration ranges with cell viability > 80% were selected for subsequent mechanistic studies: EIPA (macropinocytosis inhibitor, 60 μg/mL), CPZ (clathrin-mediated endocytosis inhibitor, 50 μg/mL), Nystatin (caveolin-dependent endocytosis inhibitor, 60 μg/mL), Brefeldin A (ER-Golgi transport inhibitor, 1 μM and 100 μM dual gradients), Monensin (Golgi transport inhibitor, 10 μM), and Nocodazole (microtubule polymerization inhibitor, 50 nM).

##### Transmembrane Transport Mechanisms of Nanoparticles

This study employed quantitative fluorescence intensity analysis to dissect the specific regulatory effects of endocytic inhibitors on cellular uptake efficiency (Figure 7D). Under basal conditions, control cells exhibited the highest fluorescence intensity (148 AU). Following treatment with specific inhibitors, all experimental groups displayed graded reductions: EIPA (5-(N-ethyl-N-isopropyl) amiloride, a macropinocytosis inhibitor) treatment significantly reduced fluorescence intensity to 100 AU, indicating macropinocytosis as the primary pathway for material uptake; Nystatin treatment decreased intensity to 119 AU, confirming a secondary contribution from caveolin-dependent endocytosis; CPZ (chlorpromazine, clathrin-mediated endocytosis inhibitor) treatment further reduced intensity to 110 AU, revealing an independent mechanistic role for clathrin-mediated endocytosis. Notably, the EIPA-treated group exhibited highly significant differences compared to all other treatments, underscoring the dominant role of macropinocytosis in the overall uptake network.

These data demonstrate that target molecule uptake results from synergistic action of multiple endocytic pathways, with efficacy following the hierarchy: macropinocytosis > caveolin-mediated > clathrin-mediated endocytosis [31].

##### Intracellular Transport Mechanisms of Nanoparticles

To elucidate the cellular uptake mechanisms of LT-NOB nanocomplexes, this study systematically investigated four specific endocytic pathway inhibitors. Quantitative analysis of fluorescence intensity changes (Figure 7E) aimed to clarify the relative contributions of distinct endocytic pathways to nanoparticle trafficking. Notably, Nocodazole (microtubule polymerization inhibitor) treatment resulted in a significant fluorescence intensity increase (181 AU), whereas Monensin (Golgi transport inhibitor), Bafilomycin A1 (lysosomal acidification inhibitor), and Brefeldin A (ER-Golgi intermediate compartment disruptor) treatments induced only moderate increases (2.78–23.61%) without statistical significance. These findings suggest limited involvement of these pathways in LT-NOB intracellular transport. The marked attenuation of nanoparticle trafficking following microtubule depolymerization indicates that microtubule-dependent pathways—such as clathrin-mediated endocytosis or macropinocytosis—constitute the primary uptake mechanisms [32]. The corresponding cellular uptake and transport pathway is illustrated in Figure 7F.

#### 3.4.4. Visualization of Nanoparticle Intracellular Transport

**Figure 8:**
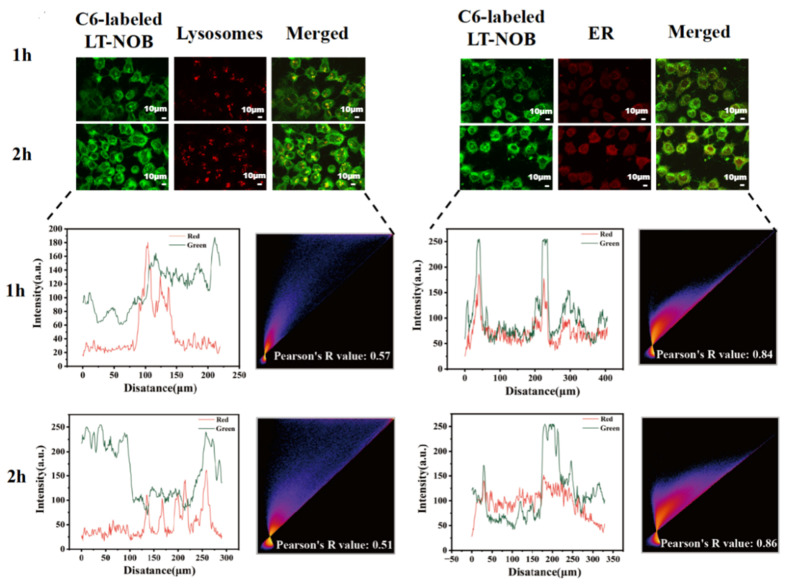
CLSM observation of colocalization of LT-NOB with lysosomes and endoplasmic reticulum, along with fitted colocalization curves and Pearson’s R value (*n* = 3). The red and green curves represent the changes in fluorescence intensity of the two dyes.

This study investigated the cellular uptake of LT-NOB nanoparticles using CLSM techniques. CLSM was employed to dynamically track their colocalization with lysosomes (labeled with LysoTracker Red) and the endoplasmic reticulum (ER) (labeled with ER-Tracker Green) (Figure 8). The results revealed that the fluorescence intensities of both lysosomes and the ER increased significantly with prolonged incubation time, accompanied by a time-dependent accumulation of nanoparticles around these organelles. This observation indicates sustained intracellular accumulation of the nanocomplexes within lysosomal and ER compartments, which may be associated with the endosomal maturation pathway and ER stress responses. As shown by the fitted curves in Figure 8, the fluorescence intensity of the nanoparticles increased more significantly over time compared to LysoTracker Red, with a Pearson’s R value of only 0.51 at 2 h. This phenomenon of “nanoparticle signal overrepresentation-colocalization decoupling” suggests that the acidic lysosomal lumen may trigger nanoparticle dissociation or structural alterations. This leads to signal decoupling between LysoTracker Red (a pH-dependent lysosomal marker) and the nanoparticles, consequently reducing long-term colocalization correlation. In contrast to the lysosomal behavior, ER colocalization exhibited “synchronized enhancement.” At the 2 h mark, the Pearson’s R value reached 0.86, and the ER fluorescence intensity closely paralleled the amplified nanoparticle signal. This strong correlation suggests that nanoparticles escaping the endo-lysosomal system may be directly trafficked to the ER via cytosolic transport, and the ER—as the central site for protein synthesis—is a critical location for cargo release.

In summary, these findings indicate a two-stage intracellular trafficking mechanism for the LT-NOB nanocomplexes: an initial sorting phase via the lysosomal pathway, followed by lysosomal escape and subsequent targeting to the ER. This dual-stage process provides crucial insights into their intracellular transport and drug delivery mechanisms [33].

#### 3.4.5. Transport Across Caco-2 Cell Monolayers

##### Calculation of Transmembrane Cumulative Permeation and Apparent Permeability Coefficient (Papp)

**Figure 9:**
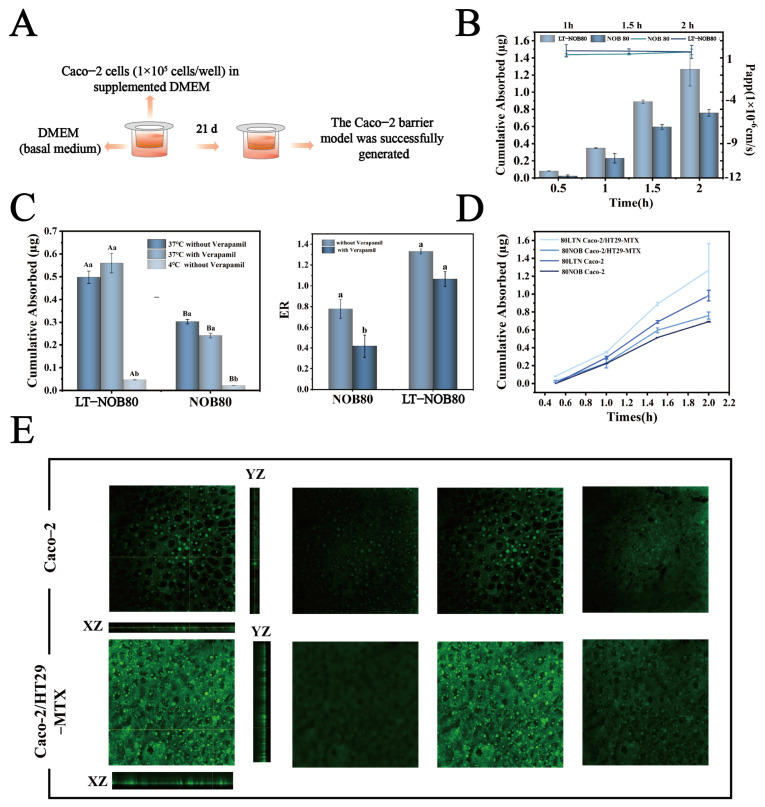
(**A**) Amount of NOB uptake and Papp values across the Caco-2 cell monolayer. (**B**) Amount of NOB uptake across the Caco-2 cell monolayer with and without verapamil and under 4 °C. (**C**) ER values with and without verapamil. (**D**) Amount of NOB uptake across the Caco-2 cell monolayer and the Caco-2/HT29-MTX co-cultured cell layer. (**E**) Penetration layer of LT-NOB across the Caco-2 cell monolayer and the Caco-2/HT29-MTX co-cultured cell layer observed by CLSM (*n* = 3). Different lowercase letters (a, b) indicate significant differences within groups, while different uppercase letters (A, B) indicate significant differences between groups (*p* < 0.05).

To evaluate the impact of the LT-NOB system on intestinal absorption characteristics, this study employed a Caco-2 cell monolayer model for in vitro permeability assays (Figure 9A). As shown in Figure 9B, the LT-NOB group and free NOB group were applied to the apical side (AP) of Caco-2 monolayers, with basal-lateral (BL) drug accumulation and apparent permeability coefficients (Papp) measured at different time points (0.5, 1, and 2 h). Results revealed that at the 2 h time point, the transmembrane cumulative permeation of NOB in the LT-NOB group reached 1.25 μg, significantly exceeding the 0.70 μg observed in the free NOB group. The Papp value for the LT-NOB group was (1.32 ± 0.15) × 10^−6^ cm/s, representing a 48.3% increase compared to the 0.89 × 10^−6^ cm/s value for the free NOB group.

These findings indicate that LT-NOB nanoparticles significantly enhance intestinal absorption efficiency of NOB through a carrier-mediated transmembrane transport mechanism. The Papp value exceeding the 1 × 10^−6^ cm/s permeability threshold demonstrates that this system effectively improves the membrane permeability of NOB.

##### Mechanistic Investigation of Transport Pathways

To elucidate the mechanistic differences in transmembrane transport between LT-NOB and free NOB, this study evaluated cellular uptake pathways through inhibitor intervention experiments and temperature sensitivity assays. As depicted in Figure 9C, the addition of verapamil (100 μM), a classic P-glycoprotein (P-gp) efflux pump inhibitor, did not significantly alter cellular uptake levels of either substrate, suggesting that P-gp-mediated active efflux processes may not participate in the transport regulation of LT-NOB or NOB. In stark contrast, reducing incubation temperature from 37 °C to 4 °C caused a 91.3% decrease in transmembrane transport of LT-NOB and a 93.3% reduction in free NOB uptake. Hypothermic treatment drastically impaired mitochondrial function, leading to about 90% decline in ATP synthesis and directly blocking energy-dependent active transport processes. Notably, while LT-NOB uptake exhibited substantial temperature-dependent reduction, its transport characteristics remained consistent with passive diffusion fundamentals—low temperatures indirectly affected drug permeation rates by altering membrane physical states (e.g., reduced fluidity due to elevated lipid phase transition temperatures) rather than relying solely on energy metabolism.

To further quantify mechanistic disparities, apparent uptake rate (ER) analysis was introduced. The LT-NOB group demonstrated an ER value of 1.3, closely aligning with the passive diffusion theoretical model (ER ≈ 1), thereby confirming its energy-independent transport process at the kinetic level. Conversely, the free NOB group exhibited a baseline ER of only 0.78, significantly deviating from theoretical expectations and indicating that its baseline transport involves energy-dependent active components. While Verapamil intervention did not alter the ER of the LT-NOB group, the NOB group ER significantly decreased to 0.39. These results directly validate the critical regulatory role of the P-gp efflux system in intracellular accumulation of free NOB, whereas the LT-NOB nanodelivery system effectively circumvents biomembrane barrier functions and metabolic inhibition constraints by establishing an energy-independent passive diffusion pathway.

In summary, fundamental differences exist between the transmembrane transport mechanisms of LT-NOB and free NOB: LT-NOB achieves efficient uptake through energy-independent passive diffusion (ER = 1.3), whereas free NOB transport significantly relies on ATP-driven active transport processes (baseline ER = 0.78, ER = 0.39 post-Verapamil treatment) [34]. In summary, fundamental differences exist between the transmembrane transport mechanisms of LT-NOB and free NOB: LT-NOB achieves efficient uptake through energy-independent passive diffusion (ER = 1.3), whereas free NOB transport significantly relies on ATP-driven active transport processes. Crucially, this passive diffusion mechanism confers a significant advantage in overcoming multidrug resistance barriers. Unlike free NOB, which serves as a substrate for P-gp and is actively pumped out of the enterocytes, the LT-NOB nanocomplex effectively ‘masks’ the drug within its core/shell structure. By preventing direct contact between the drug molecules and the P-gp efflux binding sites on the apical membrane, the nanoparticle circumvents the active efflux trap. Consequently, the absorption of LT-NOB is driven solely by the concentration gradient rather than being limited by the saturation kinetics or abundance of efflux transporters, a limitation frequently encountered in conventional drug delivery systems.

#### 3.4.6. Comparison of Permeation Behavior Between Caco-2/HT29-MTX Co-Culture and Caco-2 Monolayer Models

To more accurately simulate human intestinal physiology, this study further employed a Caco-2/HT29-MTX co-culture model (containing 90% Caco-2 intestinal epithelial cells and 10% mucus-secreting HT29-MTX cells) to investigate the transmembrane transport properties of LT-NOB. As shown in Figure 9D, the 2 h cumulative permeation of LT-NOB in the co-culture system reached 1.21 μg, exceeding the 0.89 μg observed in the Caco-2 monolayer model. CLSM 3D reconstruction results (Figure 9E) revealed significantly enhanced axial penetration depths (XZ/YZ planes) of LT-NOB in the Caco-2/HT29-MTX co-culture layer compared to the Caco-2 monolayer model. This superior penetrability can be attributed to the unique surface properties of the LT-NOB assembly. Unlike conventional cationic nanocarriers (e.g., chitosan-based systems) that are often immobilized in the superficial mucus layer due to strong electrostatic attraction with negatively charged mucin fibers, the LT-NOB nanoparticles exhibit a moderate negative zeta potential (−17 mV) and a hydrophilic polyphenol coating. These characteristics confer “mucus-inert” properties, effectively minimizing steric obstruction and adhesive entrapment. Consequently, LT-NOB achieves a deeper axial penetration depth and avoids the “mucoadhesive trap” common to traditional carriers, thereby facilitating closer contact with the underlying epithelial cells for enhanced absorption [35].

## 4. Conclusions

This study establishes a novel film-mediated nanoassembly strategy for overcoming the bioavailability challenges of hydrophobic drugs. Through systematic parameter optimization (Lys 8 mg/mL, pH 7.4, NOB 5 mg/mL), monodisperse nanoparticles with excellent dispersity (PDI = 0.03) and outstanding drug-loading capacity (drug loading = 47.25%, encapsulation efficiency = 89.5%) were successfully constructed. Under near-neutral conditions, the synergistic multivalent weak interactions between Lys and TA (encompassing electrostatic forces, hydrogen bonding, and hydrophobic effects; (Ka = 9.6 × 10^4^ M^−1^), coupled with the formation of a highly ordered β-turn-dominated secondary structure (92%), collectively endowed this system with exceptional stability. Cell experiments confirmed that LT-NOB achieves highly efficient cellular internalization primarily via a macropinocytosis-mediated pathway (38.5-fold higher fluorescence intensity), bypasses lysosomal entrapment to target the endoplasmic reticulum (Pearson’s r = 0.86), and exhibits a significantly increased apparent permeability coefficient (Papp = 1.32 × 10^−6^ cm/s) compared to free NOB. The revelation of an energy-independent passive diffusion mechanism (ER = 1.3) indicates its ability to evade active efflux barriers across biological membranes, thereby enhancing drug accumulation efficiency. Further validation using a Caco-2/HT29-MTX co-culture model demonstrated the enhanced permeation ability of LT-NOB through complex mucus layers (cumulative permeation increased by 36%), with three-dimensional imaging showing its capacity for deep drug delivery within the mucus layer. This study not only elucidates the multi-scale assembly mechanism and intracellular trafficking pathways of LT-NOB, but also provides an innovative strategy for the efficient delivery of hydrophobic drugs. The core findings—regarding the stabilization of amorphous drugs via multivalent weak interactions, the promotion of cellular uptake through macropinocytosis, the facilitation of lysosomal escape and ER targeting, and the enhancement of mucus penetration and P-gp evasion—collectively outline a versatile and generalizable platform technology. This Lys-TA nanoassembly platform holds considerable promise for adapting to and improving the bioavailability of numerous other poorly water-soluble drugs and nutraceuticals beyond nobiletin.

Crucially, the inherent design of the LT-NOB system offers a strategic advantage for bridging laboratory findings with industrial and clinical applications. Its reliance on a ‘green’, one-step aqueous assembly process aligns seamlessly with the growing industrial demand for sustainable and cost-effective manufacturing. Moreover, the exclusive use of biocompatible, naturally derived components (GRAS-grade materials) significantly lowers safety concerns and regulatory hurdles associated with synthetic carriers. These attributes establish LT-NOB not merely as a delivery vehicle, but as a versatile platform with high translational potential for developing next-generation functional foods and pharmaceutical formulations.

## Figures and Tables

**Table 1:** Detailed composition and mass ratios of the LT-NOB nanocomplex preparation under optimal and experimental conditions.

Component	Role	Stock Concentration (Cstock)	Volume Added (Vadded)	Total Mass Input (Minput)	Remarks (Experimental Range)
Nobiletin (NOB)	Functional Cargo (Core)	5 mg/mL	1.0 mL	5.00 mg	Fixed at 5 mg/mL during Lys optimization; varied (4–7 mg/mL) in drug conc. studies.
Tannic Acid (TA)	Crosslinker/Shell	40 mg/mL	20 μL	0.80 mg	Fixed concentration and volume throughout the study.
Lysozyme (Lys)	Wall Material /Shell	8 mg/mL (Optimal)	40 μL	0.32 mg	Stock concentration varied from 2 to 12 mg/mL (corresponding to 0.08–0.48 mg mass input).
Buffer (MOPS)	Solvent Medium	0.01 M (pH 7.4)	9.0 mL	-	pH varied from 5.0 to 8.0 during optimization.

**Table 2:** Comparison of physicochemical properties of LT-NOB nanoparticles under optimal and suboptimal synthesis conditions.

Parameters	Optimal Conditions	Suboptimal Conditions (Deviations)	Impact of Deviation
Synthesis	Lys: 8 mg/mL pH: 7.4 NOB: 5 mg/mL	Lys: <8 mg/mL or >8 mg/mL pH: Acidic (<7.0) or Alkaline (>8.0) NOB: Deviated from 5 mg/mL	Lys Deviation: Reduced encapsulation efficiency (EE%) and drug loading (DL%). pH Deviation: Weakened electrostatic or hydrophobic interactions.
Variables Particle Size (Diameter)	212 nm (Uniform distribution)	Significantly Increased or Broadened distribution	pH deviation leads to aggregation or large precipitates due to reduced binding affinity.
Polydispersity Index (PDI)	0.03 (High monodispersity)	>0.3	Indicates poor dispersion and system instability.
Zeta Potential	−17 mV	Varies (e.g., reduced negative charge magnitude)	Less stable colloidal system prone to aggregation.
Encapsulation Efficiency (EE%)	89.50%	<89.5%	Lower Lys concentration results in insufficient wall material; extreme pH disrupts assembly forces.
Drug Loading (DL%)	47.25%	Lower	Excessive Lys (12 mg/mL) increases shell thickness without adding drug payload.
Morphology and Crystallinity	Regular spherical shape; Amorphous NOB (Crystallization suppressed)	Irregular shape; Crystalline precipitates observed	Appearance of birefringence in POM indicates failure to inhibit NOB crystallization.

## Data Availability

The original data and analytical results presented in this study are included in the article and its Supplementary Materials. For further inquiries, please contact the corresponding author.

## Supplementary Materials

The following supporting information can be downloaded at: https://www.mdpi.com/article/10.3390/biom16020242/s1, Figure S1. TEM, CLSM, and POM images of LT-NOB at different Lys concentrations. Figure S2. TEM, CLSM, and POM images of LT-NOB at different pH. Figure S3. TEM, CLSM, and POM images of LT-NOB at different NOB concentrations. Figure S4. MTT Cytotoxicity Assay. Figure S5. QCM-D. Figure S6. TEM-NOB. Figure S7. Gating strategy for flow cytometry. Figure S8. Particle size distribution chart and CLSM.
