# Supplementary material for: Membrane-Mediated Nanoassembly of Lysozyme–Tannic Acid for Crystallization-Suppressed Nobiletin Delivery: Enhanced Cellular Uptake and Mucus Penetration
Source: Biomolecules. 2026 Feb 3;16(2):242. doi: 10.3390/biom16020242 (PMC12938187; doi:10.3390/biom16020242)
Supplement: Supplementary file 1 [file biomolecules-16-00242-s001.zip › biomolecules-4067117-supplementary.pdf]

biomolecules

MDPI

Article

Membrane-Mediated Nanoassembly of Lysozyme–Tannic Acid for Crystallization-Suppressed Nobiletin Delivery: Enhanced Cellular Uptake and Mucus Penetration

Hongyu Liang [1], Jiahao Xing [2], Qiuyue Hou [1], Luyang Bao [1], Bin Li [2,3], Bin Zhou [1,*] and Hongshan Liang [2,3,*]

[1] Cooperative Innovation Center of Industrial Fermentation (Ministry of Education & Hubei Province), School of Life and Health Sciences, Hubei University of Technology, Wuhan 430068, China
[2] College of Food Science and Technology, Huazhong Agricultural University, Wuhan 430070, China
[3] Key Laboratory of Environment Correlative Dietology (Huazhong Agricultural University), Ministry of Education, Wuhan 430070, China
* Correspondence: zhoubin4111@163.com (B.Z.); lianghongshan@mail.hzau.edu.cn (H.L.)

Supplementary Methods

Preparation of the LT-NOB system under different conditions.

1.1. System Optimization under Different pH Conditions

Based on the optimal Lys concentration screened in Section 2.2.1, the pH-dependent behavior of the ternary complex system was further evaluated. Experiments created differentiated microenvironments by adjusting the pH (5.0, 6.0, 7.0, 7.4, 8.0) of the MOPS buffer system. The preparation steps for the ternary complex were strictly consistent with the conditions described in Section 2.2.1.

1.2. System Optimization with Different NOB Content

To investigate the maximum drug-loading capacity of the Lys-TA complex for nobiletin (NOB), the encapsulation performance was systematically evaluated by gradient adjustment of the initial NOB concentration (4–7 mg/mL), based on the optimal Lys concentration and pH conditions determined in Sections 2.2.1–2.2.2. The preparation steps for the ternary complex remained consistent with Section 2.2.1.

 https://doi.org/10.3390/biom16020242

Supplementary Figures

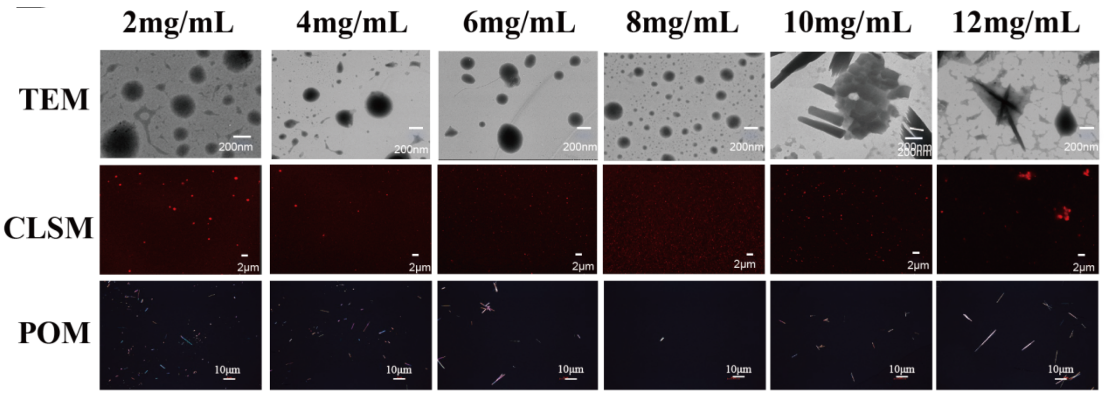

Figure S1. TEM, CLSM, and POM images of LT-NOB at different Lys concentrations.

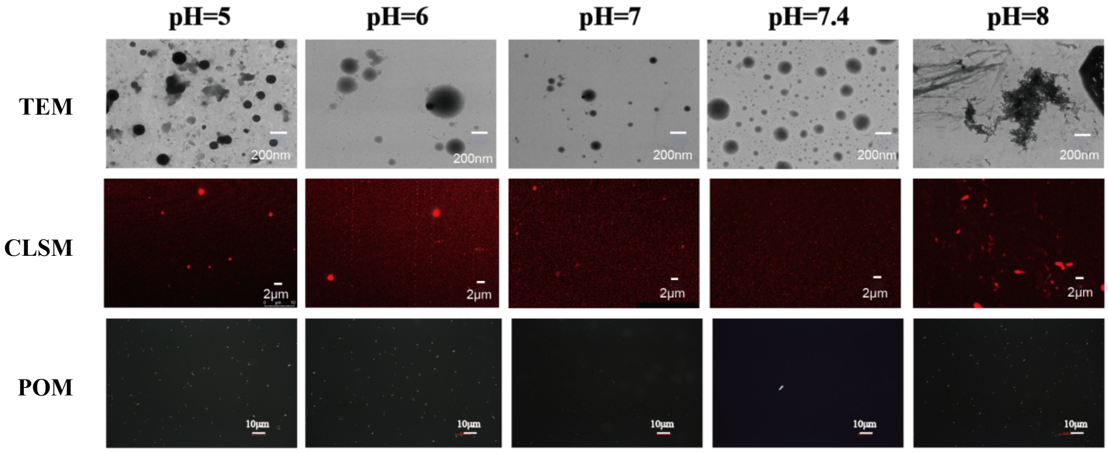

Figure S2. TEM, CLSM, and POM images of LT-NOB at different pH.

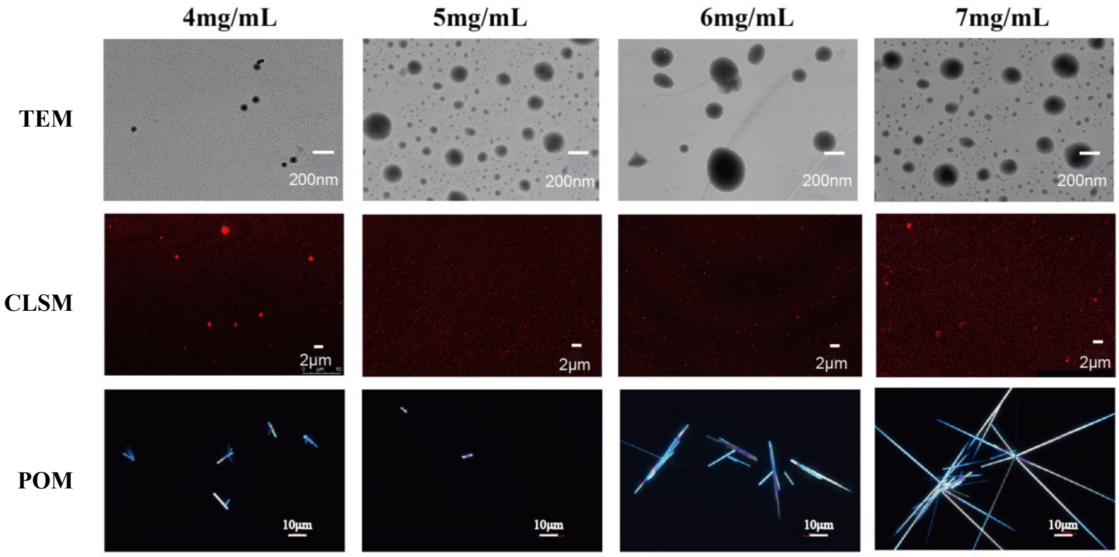

Figure S3. TEM, CLSM, and POM images of LT-NOB at different NOB concentrations.

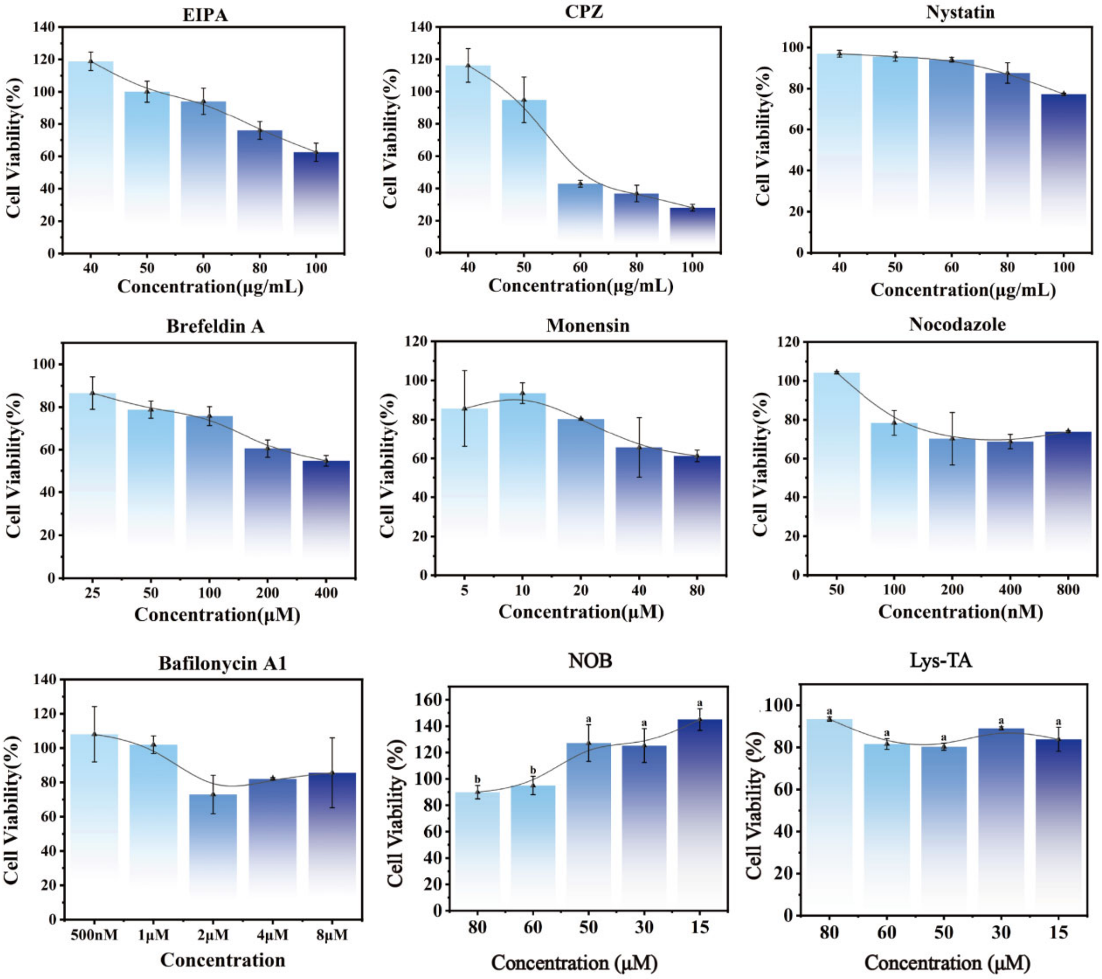

Figure S4. MTT Cytotoxicity Assay.

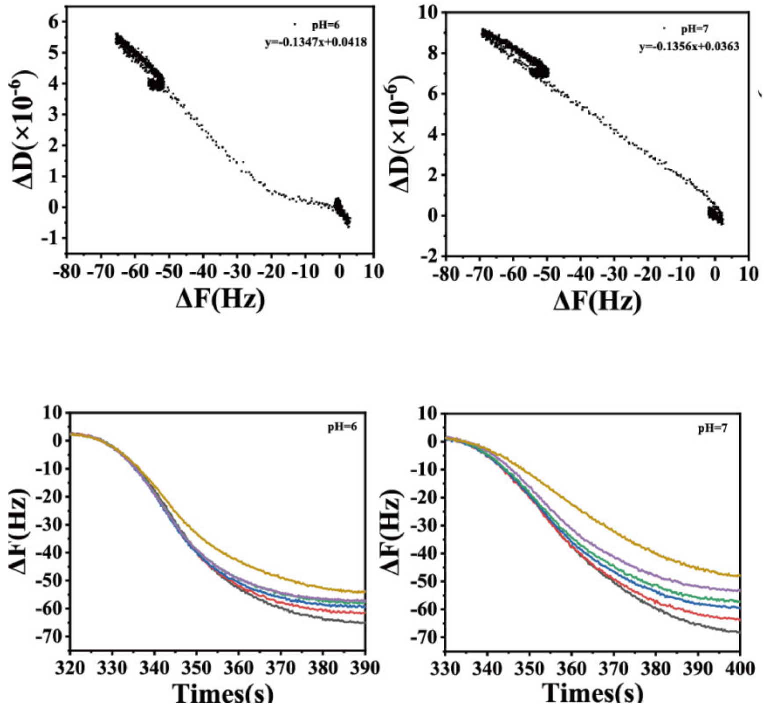

Figure S5. QCM-D.

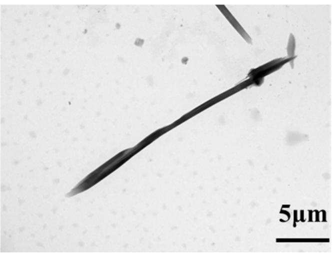

Figure S6. TEM-NOB.

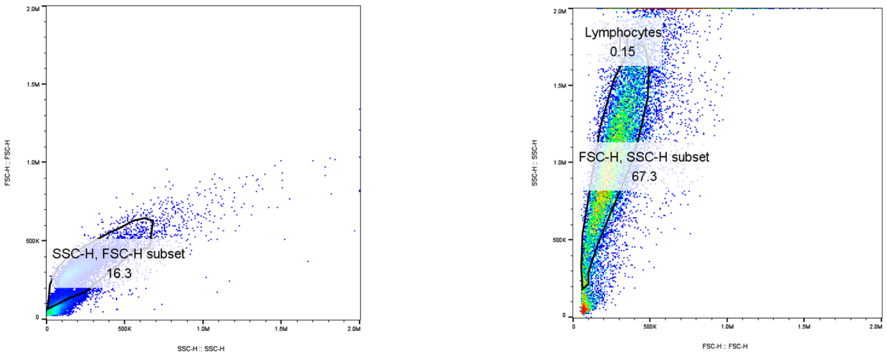

Figure S7. Gating strategy for flow cytometry.

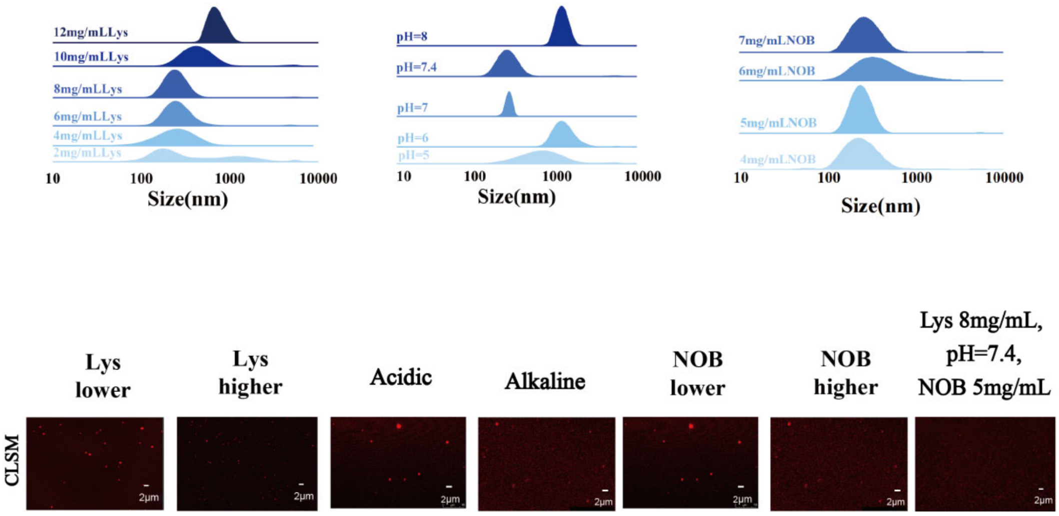

Figure S8. Particle size distribution chart and CLSM.

https://doi.org/10.3390/biom16020242
